# Spatio-Temporal Regulation of PKC Isoforms Imparts Signaling Specificity

**DOI:** 10.3389/fimmu.2016.00045

**Published:** 2016-02-17

**Authors:** Arkajyoti Mukherjee, Sayoni Roy, Bhaskar Saha, Debasri Mukherjee

**Affiliations:** ^1^National Centre for Cell Science, Pune, India

**Keywords:** PKC isoforms, spatio-temporal regulation, subcellular distribution, PKC-signaling module, intracellular pertubators

## Introduction

**Figure 1:**
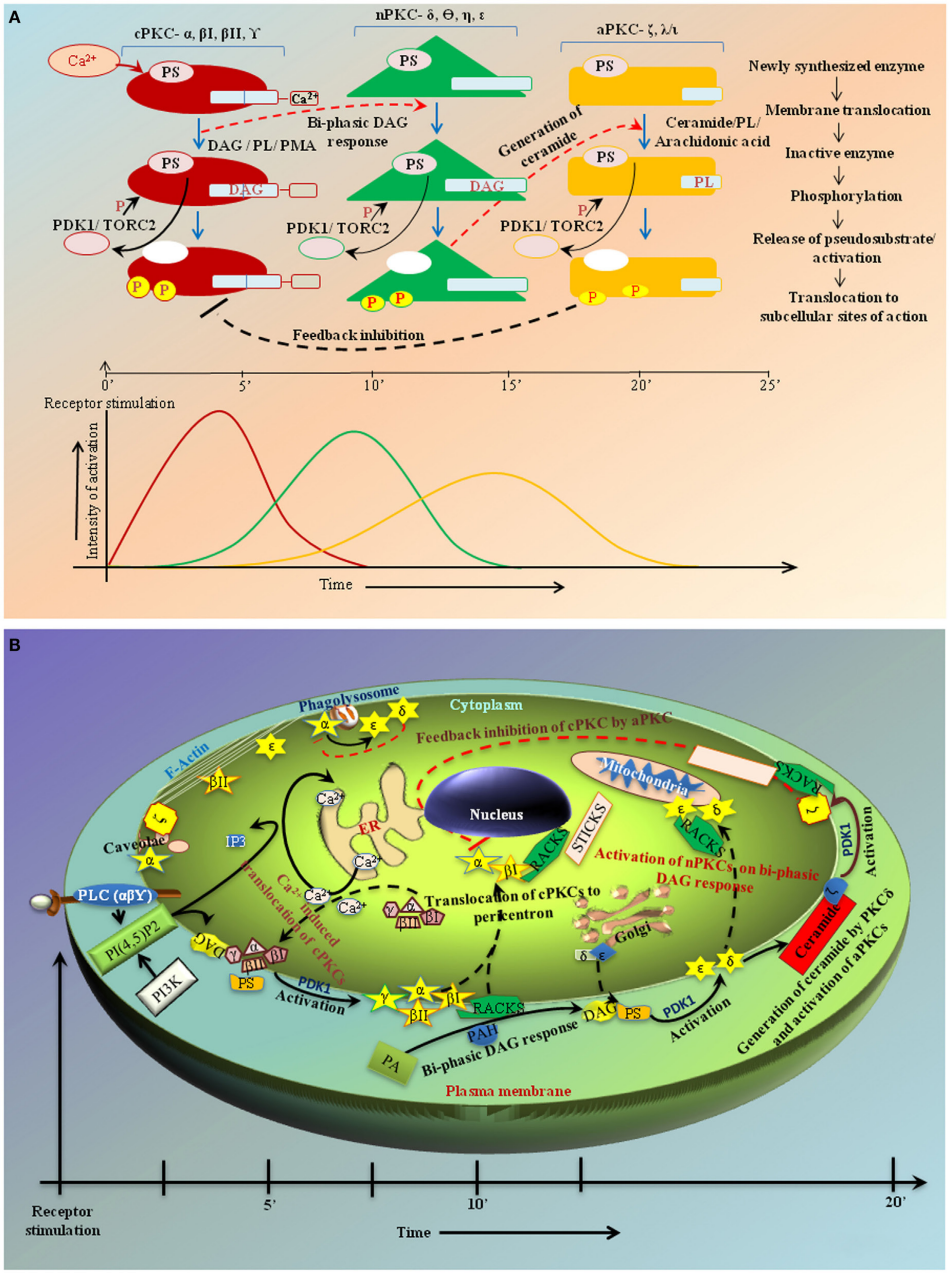

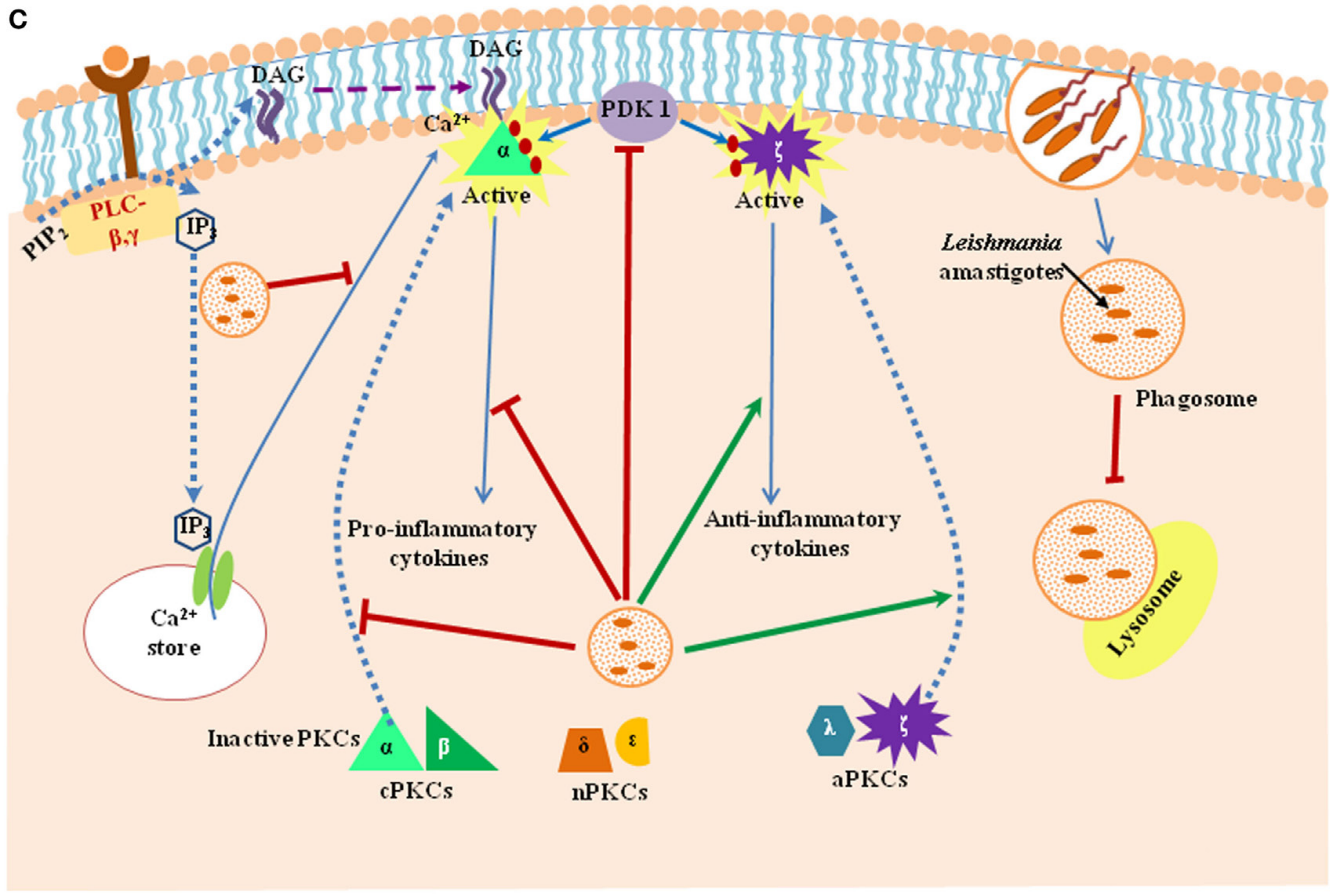
**(A)** Hypothetical figure showing sequential activation of the PKC isoforms in response to receptor-stimulus coupling. Analyses from various studies indicate that in cases where a single stimulus activates multiple isoforms of different classes the classical PKC isoforms are usually activated first and to the greatest degree followed by the novel and atypical forms. **(B)** Kinetics of activation and subcellular distribution of PKC isoforms. The various classes of PKC isoforms are localized at different subcellular sites both prior and post activation. They are translocated to the membrane for activation in response to their respective cofactors. Post activation, the isoforms are translocated to specific subcellular locations by their binding partners, which bring them close to their respective substrates. DAG, diacylglycerol; IP3, inositol tri-phosphate; PA, phosphatidic acid; PAH, phosphatidic acid hydrolase; PI3K, phosphatidyl inositol-3-kinase; PLC, phospholipase C; PKC, protein kinase C; PhosIns(4,5)P2, phosphatidyl-inositol-4,5-bis phosphate; RACK, receptors for activated C-kinases; STICK, subtrates that interact with C-kinases. **(C)** Modulation of PKC isoforms by *Leishmania* infection. The figure shows modulation of PKC isoforms by *Leishmania* as it enters a host cell, for instance, macrophages, by preventing phagosome maturation. It interferes with the translocation of isoforms to membranes and inhibits PDK-1. It inhibits Ca^2+^ efflux to prevent DAG-mediated activation of cPKCs α and β, which are involved in proinflammatory cytokine production thus resulting in disease progression concomitantly enhancing PKCδ- and ζ-mediated production of anti-inflammatory cytokines to suppress host immune response conducing in parasite survival.

Nishizuka and colleagues discovered protein kinase C (PKC) as a calcium-dependent, lipid cofactor-sensitive protein kinase (1). Initially known as the only physiological effector for tumor-promoting phorbol esters, this ubiquitous enzyme eventually took center stage in cellular signaling. Part of the AGC kinase branch of the kinome, PKC is a family of serine/threonine kinases comprising 11 isoforms encoded by 9 genes and grouped into 4 classes – classical (cPKCs-α, βI, βII, γ), novel (nPKCs-δ, ϵ, η, θ), atypical (aPKCs-ζ, ι/λ), and PKCμ (a form between novel and atypical isoforms) (Box 1). Their considerable structural homology, overlapping substrate specificities, and biochemical properties indicated at least partial enzymatic redundancy and rendered the task of identifying isoform-specific functions challenging. The initial phase of PKC research correlated the unique structural features of the isoforms with their functions (Box 1) (2). But, with their structural overlaps and hugely varying functions in different models, specificity of the isoforms became a confounding puzzle demanding stringent isoform-specific regulation to avoid functional redundancy. To define this stringency, subsequent PKC research focused on the upstream and downstream regulatory mechanisms (3, 4), highlighting the importance of subcellular distribution as a function of time (5). As simultaneous activation of all PKC isoforms would be energetically and spatially conflicting for decoding the message received by the cell surface receptor, cell type, and stimulus-specific selective and sequential activation deemed justified (6). The recent phase of PKC research propounds a PKC-signaling module (7, 8) wherein an inter-isoform network regulates the PKC isoforms’ activity (Box 2; Figure 1B). Here, we propose activator- and cofactor-specific sequential activation of PKC isoforms in a spatio-temporal model wherein selective subcellular compartmentalization quantitatively determines the isoform-specific effector functions.

Box 1 **Structural features of PKC isoforms defining functional specificity.**

PKC serve as a paradigm for the reversible regulation of membrane localization by the concerted action of two membrane-targeting modules.

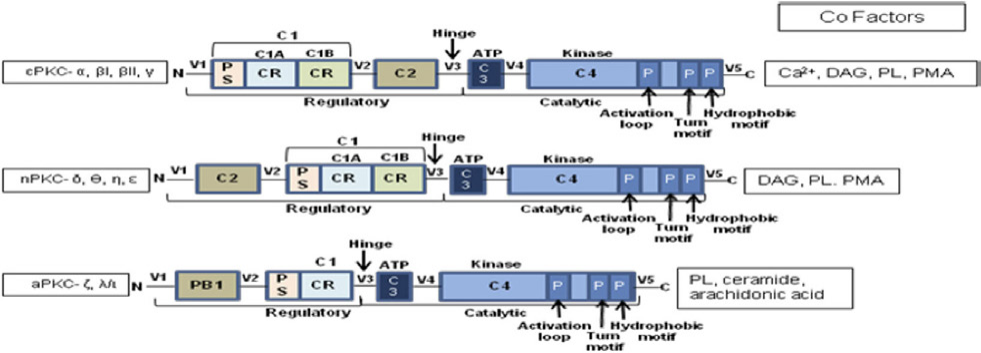

C1: a cysteine-rich region of approximately 50 residues present in all PKC isozymes. In cPKCs and nPKCs, it is present as a tandem repeat C1A and C1B. aPKCs contain a single copy of the domain termed atypical because it does not bind phorbol esters. The domain contains two pulled apart β-sheet forming the ligand-binding pocket. Two zinc atoms are coordinated by two histidines and six cysteines at opposite ends of the primary sequence stabilizing the domain. In aPKCs, one face of the ligand-binding pocket is compromised, so that the module cannot bind phorbol esters or DAG.

Ligand binding dramatically alters the surface properties of the module (C1B). The ligand caps the hydrophilic ligand-binding pocket, so that the top-third of the C1 domain presents a continuous hydrophobic surface thus achieving membrane targeting by simply altering membrane properties.

C2: an independent membrane-targeting module that binds calcium in cPKCs, but not in nPKCs. C2 domain is a β-strand rich globular domain with loops formed by sequences at opposite ends of the primary structure. Two topological variants exist – Type I for domains that follow the C1 domain (cPKCs) and Type II for domains that precede C1 (nPKCs). In the calcium-responsive C2 domains of cPKCs, the pocket is lined by multiple aspartic acid residues that coordinate two to three calcium ions that act as a bridge between the C2 domain and the phospholipid head groups of the membrane.

CR, cysteine rich; PS, pseudosubstrate; DAG, diacylglycerol; PMA, phorbol-12-myristate 13-acetate; PB1, Phox–Bem 1; C, constant regions; V, variable regions.

Box 2 **Chronological epochs of PKC research.**

*1977–1987: introducing PKC*

• Discovery of PKC by Nishizuka and colleagues (1).
• Structural analysis of the PKC isoforms.
• Takai et al. showed PKC to be reversibly activated by Ca^2+/^phospholipid and DAG (9).
• Identification of PKC as a target for phorbol ester class of tumor promoters by Castagna et al. (10).
• Discovery of the pseudosubstrate region by House and Kemp (2).
• 1988–1997: the decade of elucidation of PKC structure–function relationship
• Elucidation of the structural basis of PKC function.
• Identification of receptors for activated C-kinases by Mochly-Rosen and coworkers (11).
• First report by Nishizuka about the role of PKC in cellular signaling (3).
• In 1994, Dekker and Parker for the first time raised the question of specificity of PKC isoforms (4).
• Cloning of first RACK by Mochly-Rosen et al. (12).
• Role of anchoring protein in localization of PKCs (5).
• For the first time, the role of binding proteins in PKC isoform-specific functions was suggested.

*1998–2007: the decade of studies on the spatio-temporal regulation of PKC isoforms*

• Identification of PKC anchoring proteins as a means for isozyme selectivity by Mochly-Rosen and colleagues in 1998 (13).
• Detection of substrates that interact with C-kinases (STICKs) through overlay assay by Jaken (14).
• Compartmentalization of PKC through binding proteins and substrates.
• Studies on the temporal kinetics of PKC function.
• Elucidation of cell and stimulus-specific actions of PKC isozymes.

2008–present: inter-PKC regulation and PKC-signaling module

This is the decade where the concept of inter-PKC regulation in calibrating receptor triggered effector functions is gaining popularity leading to the possible build-up of a PKC-signaling network in space-time coordinates.

## Structural Bias to PKC Function?

Structural reconfigurations of the PKC isoforms play a central role in orchestrating their spatial distribution and activation (15). C1 domain ligands – diacylglycerol (DAG) and phosphatidylserine (PS) – recruit PKC by altering the surface properties of the domain to favor membrane penetration. Studies on GFP–PKC with the fluorescent phorbol ester analog sapintoxin-D showed differential subcellular localization of C1 ligands determining subcellular targeting of PKCs (16). The nPKC C1B domain has a 100-fold higher affinity for DAG compared to cPKCs due to an invariant tryptophan residue at position 22 as opposed to a tyrosine in case of cPKCs resulting in rapid plasma membrane localization for cPKCs versus slower and sustained Golgi localization for nPKCs (17). The C2 domain functions as a Ca^2+^-regulated membrane anchor in cPKCs (6, 15) while regulating protein–protein interactions and spatial distribution through phosphotyrosine-binding modules in nPKCs (18, 19). The aPKCs are not responsive to either DAG or Ca^2+^ and instead possess a Phox–Bem (PB)1 domain that facilitates interactions with scaffolding proteins leading to constitutive activation (20). Phosphorylation plays a key role in determining the cellular levels of PKC rendering them catalytically competent and protecting them from degradation (17). Classical and novel PKCs are constitutively phosphorylated at three conserved residues: the activation loop – the first rate-limiting step governed by phospho-inositide-dependent kinase (PDK)-1 that aligns residues within the active site for catalysis, followed by phosphorylation at the turn motif and autophosphorylation of the hydrophobic loop. For cPKCs and nPKCs, these phosphorylation events require mTORC2. Phosphorylation at the hydrophobic motif controls the stability of the enzyme promoting degradation on dephosphorylation (17). A phosphomimetic glutamic acid prevents phosphorylation at the hydrophobic motif of aPKCs. Although catalytically competent, these phosphorylated PKCs remain inactive in the cytosol due to binding of the pseudosubstrate to its kinase domain (Figure 1A) until appropriate cofactor interaction provides the necessary energy to expel the pseudosubstrate and activate PKC. So, the key to functional specificity *in vivo* may lie in the isoforms’ primary structure and the cofactors governed conformational plasticity. Yet, their promiscuity *in vitro* suggests involvement of factors beyond the primary structure leading to the studies on the spatio-temporal regulation of these isozymes.

## PKC Isoform Functions in Space-Time Coordinates

Functional specificity of the PKC isoforms depends on the proximity to their substrates in specific intracellular compartments effected through multiple binding proteins that also determine the precise duration and amplitude of PKC activity thus providing a mechanism for integrating PKC-mediated signaling with other cellular activities. PKCβI interacts with Bruton’s tyrosine kinase to positively regulate JNK signaling and cytokine gene expression in mast cells (6, 21). Partition defective-3 (PAR3) protein interacts with PKCζ to activate pathways leading to embryonic polarity and asymmetric cell division (6, 22), whereas PAR4 inactivates PKCζ leading to apoptosis (6, 23). Ras-related nuclear protein-binding protein-9/10 integrates PKCδ and γ signals to dictate efficient regulation of dopaminergic D1 receptor signaling (24). In CHO cells, initial phorbol 12-myristate 13-acetate (PMA) treatment translocates and colocalizes receptor for activated C-kinases (RACK) 1 and PKCβII to the cell periphery that later move to the perinuclear area (25). Thus, RACK–PKC complex can move from one cellular site to another likely resulting in different molecular events at each site depending on the available substrate. PKCϵ interacts specifically with filamentous actin through a binding site located between its two cysteine-rich regions to enhance glutamate exocytosis from nerve terminals (26). Notwithstanding the considerable stimulus and cellular variability in their subcellular distribution, certain PKC isoforms exhibit unique localizations. PKCθ is redox dependently recruited to the plasma membrane of naive T-cells (27). The scaffolding protein A-kinase anchor protein (AKAP) 450 (25) associates with nascent PKCϵ within Golgi/centrosome membranes and dissociates on maturation of PKCϵ (17). PKCζ shows a remarkable range of functions reflecting its multiple cellular locations and interacting partners (28). Interactions with binding partners sometimes also affect PKC pharmacological profile like AKAP-79 protecting PKC from certain ATP competitive inhibitors and altering the susceptibility of PDK-1 to ATP analog inhibitors (29). These observations indicate that the structural features determine the isoforms’ translocation and activation pattern, whereas the binding proteins contribute plasticity and specificity.

As multiple PKC isoforms may be activated by a single stimulus, it is logical to assume that differential activation dynamics can impose specificity. Following receptor ligation, the isoforms exhibited differences in activation kinetics and subcellular localization. The kinetics of Ca^2+^-induced translocation triggered by the C2 domain is faster than that triggered by the C1 domain (30) (Figure 1A) indicating a possible sequential activation pattern (31) guided by stimulus specificities in many cell types including macrophages and T-cells (8, 31). Indeed, studies in chick muscle cells have shown short-term stimulation to initially trigger PKCα/β membrane translocation followed by PKCδ to a lesser degree (32). PKCδ-activated acid sphingomyelinase cleaves sphingomyelin releasing ceramide that activates PKCζ and inactivates the cPKCs (15). These observations support the “sequential PKC isoform activation” hypothesis and an inter-isoform regulation (Figure 1A).

## Inter-Isoform Regulation and Their Sites of Perturbation

Coordination between the concomitantly expressed and activated PKC isoforms within a temporal framework seems to be regulated by transphosphorylation of the isoforms (33). Following receptor stimulation, classical isoforms are acutely activated as the release of calcium is fast and transient. Calcium sequestration into the endoplasmic reticulum is also rapid (Figure 1B), so deactivation of the classical isoforms likely occurs earlier than the others. Studies have noted a feedback inhibition of cPKCs on activation of novel and atypical isoforms (8, 15). PKCδ and ϵ have opposing effects in multiple pathological conditions, including cardiac ischemia, cancer, apoptosis, and cell proliferation (34). During endosome formation in phagocytic cells, the initial calcium burst activates the PKCα leading to initiation of phagosome formation (35). This is sequentially followed by translocation and activation of PKCδ and ϵ that play a role in phagosome maturation and lysosomal fusion (36). PKCα can phosphorylate PKCϵ, so it is possible that the effects of PKCα are shared by PKCϵ (37). Ceramide generated by PKCδ activates PKCζ that inhibits PKCα/βII at the perinuclear space (15, 38). PKCζ has constitutive kinase activity that can activate other PKC isoforms. Sequential activation of PKC isoforms thus explains the dynamic modulation of cellular responsiveness dictated by the strength of stimulus (Figure 1B). Following TCR activation, PKCα acts upstream of PKCθ to activate NFκB (39). Inhibition of PKCθ abrogates the PKCα response indicating presence of a feedback loop between the isoforms or, as proposed here, a sequential activation of PKCα and PKCθ. Conversely, PKCθ and PKCβ seem to have physiologically redundant roles in TCR/CD28-dependent NFκB and NFAT transactivation in primary mouse CD3^+^ T cells (40). In intestinal epithelium, PKCα downregulates while PKCϵ upregulates cyclin D1 thus contributing to the opposing effects of these isoforms in tumor progression (41). nPKC isoforms are sequentially recruited to the immunological synapse with PKCϵ and η being recruited first followed by PKCθ (42). Opposing effects of PKCη and PKCθ on relative numbers of CD4^+^ and CD8^+^ T cells have been observed in mice (43). PKCα and PKCβ cooperate functionally in CD3-induced *de novo* IL2 mRNA transcriptional transactivation in primary mouse T cells independently of the actions of PKCθ (44). PKCϵ acts upstream of PKCα in the signal transduction of ischemic preconditioning of human myocardium (45). These inter-PKC regulations through feedback loops and sequential activation constitute a functioning PKC module. Mechanistically, an adaptor with dynamically controlled multiple scaffolds may connect one PKC isoform to the next.

## Differential PKC Isoform Regulation in Infection

With so many available isoforms, PKC is an ideal candidate for intracellular perturbators (Figure 1C). Reciprocal action of PKC isoforms has been observed in many infection and disease models. Histone deamination in neutrophils during pathogen infection or chronic inflammation is activated by PKCζ while PKCα inhibits it (28). In bone marrow-derived mast cells (BMMs), PKCα and θ positively regulate IL6 and TNFα production against filarial nematode *Acanthocheilonema viteae* infection, whereas PKCβ and ϵ act as negative regulators (46). cPKC activation seems to be associated with proinflammation as evident by the activation of these isoforms on coincubation with IFNγ (47). In *Mycobacterium tuberculosis*-infected macrophages, PKCα upregulates proinflammatory response in conjugation with TLR2 on pretreatment with arabinosylated lipoarabinomannan (48). While PKCα/β mediates CD40-induced p38MAPK phosphorylation and IL-12 expression, PKCδ and ζ inhibit it reciprocally by enhancing ERK1/2 phosphorylation and IL-10 production (8). PKCα degrades periphagosomal F-actin required for phagosomal maturation (49). This is key to the survival or elimination of the pathogens, which are either phagocytosed or internalized *via* receptor-mediated endocytosis. Infection might cause impairment of Ca^2+^-host signal transduction, which in turn may affect classical PKC isoforms. *Leishmania donovani* infection or recombinant IL-10 treatment of macrophages inhibits both the activity and expression of the cPKC isoforms (50). *Leishmania major* also impairs PKCα, βI, βII isoforms while enhancing PKCδ and ζ isoforms in macrophages (8, 37). Increased generation of membrane ceramide (51) and concomitant cholesterol extrusion (52) may cause the inhibition of PKCα/β and activation of PKCζ in macrophages during *Leishmania* infection (8). Comparison between DAG and ceramide elucidates the different kinetics of aPKC isoforms from the other two. Ceramide activates the alternate signaling pathways leading to anti-inflammatory responses. PKCζ has been shown to be involved in the activation of arginase I, the enzyme responsible for inhibition of iNOS and inflammatory responses toward parasites (53). Intracellular pathogens can interfere with any of these mechanisms in order to tune the PKC-mediated signaling pathways according to their convenience (Figure 1C). Being least specific in regards to cofactor and activator requirements, nPKCs play a dual role in inflammation (8, 36, 47, 54). So, the PKC isoforms and the inter-isoform regulation might serve as targets for cellular signaling modulation for therapeutic intervention against pathophysiological conditions.

## Conclusion

From the evidences gathered, the existence of inter-PKC regulation and a PKC-signaling module seem a logical plausibility. The signaling specificity is generated by the combinatorial assemblies and spatio-temporal dynamics of the PKC isoforms allowing calibration and kinetic modulation of the pathways of the receptor-regulated cellular responsiveness. Although the extreme diversity of PKC responses based on cellular and stimulus differences and the lack of appropriate tools and specific inhibitors pose a major hurdle in building of a generic PKC-signaling map, the analyses provide a conceptual framework placing all PKC isoforms in a single space-time network and a novel principle for devising therapeutic strategies against pathophysiological conditions.

## Author Contributions

AM and SR wrote the manuscript and built the figures. DM and BS critically reviewed the manuscript and made the necessary edits. The final manuscript was a result of the joint efforts of all the authors.

## Conflict of Interest Statement

The authors declare that the research was conducted in the absence of any commercial or financial relationships that could be construed as a potential conflict of interest.
